# Adherence to perioperative antimicrobial prophylaxis in cancer surgeries: A single-center retrospective study from Oman

**DOI:** 10.1016/j.ijregi.2026.100845

**Published:** 2026-01-16

**Authors:** Bassem Awada, Manal Al-Hatrushi, Amal Abdallah, Lamia Alnor, Hasan Al-Sayegh, Juma AlKasbi, Amna Al-Hashar

**Affiliations:** 1 Infectious Diseases Division, Internal Medicine Department, Sultan Qaboos Comprehensive Cancer Center, University Medical City, Muscat, Sultanate of Oman; 2 Pharmacy Department, Sultan Qaboos Comprehensive Cancer Center, University Medical City, Muscat, Sultanate of Oman; 3 Medical Oncology Division, Sultan Qaboos Comprehensive Cancer Center, University Medical City, Muscat, Sultanate of Oman; 4 Research Laboratories Department, Sultan Qaboos Comprehensive Cancer and Research Center, University Medical City, Muscat, Sultanate of Oman; 5 Surgical Oncology Department, Sultan Qaboos Comprehensive Cancer Center, University Medical City, Muscat, Sultanate of Oman

**Keywords:** Antimicrobial stewardship, Preoperative care, Surgical wound infection, Neoplasms

## Abstract

**Objectives:**

Surgical antimicrobial prophylaxis (SAP) is essential for preventing surgical site infections (SSIs), particularly in oncology patients. However, adherence to SAP guidelines globally remains inconsistent. This study evaluated adherence to SAP protocols at a tertiary oncology center and assessed associated factors with non-adherence and SSI outcomes.

**Methods:**

A retrospective review was conducted at Sultan Qaboos Comprehensive Cancer and Research Center (SQCCCRC) from August 2022 to July 2023. SAP prescriptions were evaluated for four criteria: antibiotic choice, weight-based dosing, re-dosing, and duration. Non-adherence to any of the four components considered non-adherent. Demographics, cancer type, surgical procedure, wound classification, procedure duration, SSIs, and microbiology results were extracted from patient records. We used bivariate analysis and multivariable logistic regression model to identify factors associated with non-adherence. The cumulative incidence of SSIs was also calculated.

**Results:**

Of the 261 surgeries reviewed, overall adherence to SAP protocols was 65%. Weight-based dosing adherence was highest (99.6%), while re-dosing adherence was lowest (80%). Univariate analyses showed non-adherence linked to higher patient weight (*P* = 0.041), male gender (*P* = 0.01), wound classification (*P* < 0.001) and longer surgery duration (*P* < 0.001), while breast surgery had better adherence (*P* = 0.0025). In the multivariable model, non-adherence was independently associated with gyne-oncology procedures (odds ratio [OR] = 7.76, 95% confidence interval [CI] = 2.13-30.86; *P* = 0.002) and longer surgery duration (OR = 1.35 per hour, 95% CI = 1.16-1.58; *P* = 0.0001). The cumulative incidence of SSIs was 8.4%, comprising 52% organ-spaced, 33.3% deep SSI, and 14% superficial SSIs. Non-adherent cases were more likely to have SSI compared to adherent (15.7% vs 4.7%, *P* = 0.004).

**Conclusions:**

The study found suboptimal overall adherence to SAP protocols, particularly in re-dosing practices. Targeted antimicrobial stewardship interventions are warranted to address these gaps and minimize infection risk, especially in high-risk oncology populations.

## Highlights

• Surgical antimicrobial prophylaxis adherence was suboptimal (65.9%) in cancer surgery.
• Weight-based dosing showed near-universal compliance.
• Antibiotic choice, duration, and re-dosing remained suboptimal.
• Prolonged procedures warrant targeted stewardship efforts.

## Introduction

Surgical site infections (SSIs) are the most common healthcare-associated infections among patients undergoing surgery [1]. They are associated with increased morbidity, mortality, and healthcare costs [1]. In the surgical oncology population, SSIs carry an even greater burden due to the complexity of procedures, immunosuppression, and postoperative delays in cancer treatment [2]. Effective perioperative antibiotic prophylaxis, when administered according to established guidelines, remains a cornerstone intervention for reducing the risk of SSIs [3]. However, despite the availability of international guidelines, several reports have documented poor adherence [4].

Given the heightened vulnerability of oncology patients and the complexity of oncologic surgeries, evaluating compliance with surgical antimicrobial prophylaxis (SAP) is essential for improving clinical outcomes. This study aimed to assess adherence to SAP guidelines at the Sultan Qaboos Comprehensive Cancer Care and Research Center (SQCCCRC), Oman’s national tertiary oncology center. Secondary objectives included examining the association between SAP non-adherence and identifying patient- and surgery-related factors linked to non-compliance.

## Methods

### Study design

We conducted a retrospective cohort study at the Sultan Qaboos Comprehensive Cancer Care and Research Center-University Medical City (SQCCCRC-UMC), a 120-bed tertiary cancer center in Muscat, Oman, specializing in the management of solid tumors. The study period spanned from August 1, 2022, to July 31, 2023. The study received the ethical approval from the SQCCCRC-UMC Institutional Review Board (CCCRC-52-2023).

### Study population

All oncology patients aged >18 years who underwent surgeries were included. Those who had pre-existing infection at the time of surgery were excluded. The primary objective of our study was to evaluate overall adherence to local institutional SAP guidelines, which are adopted from international recommendations issued by the Infectious Diseases Society of America (IDSA) and the American Society of Health-System Pharmacists (ASHP) [5]. Adherence was assessed based on the following criteria: (1) appropriate selection of antimicrobial agent according to guidelines, (2) correct weight-based dosing adjustments, (3) appropriate timing of intraoperative re-dosing, and (4) appropriate duration of postoperative antibiotic therapy. The duration of antibiotic therapy was considered appropriate if antibiotics were prescribed for no more than 24 hours postoperatively.

### Statistical analysis

Descriptive statistics were used to summarize patient demographics, surgical characteristics, and adherence rates to SAP guidelines. Categorical variables were reported using frequencies and percentages, while continuous variables were summarized using means and standard deviations.

To evaluate the relationship between overall ASP adherence and risk factors, the chi-squared test or Fisher’s exact test were used for categorical comparisons. For continuous variables, we used the Wilcoxon rank-sum test. Univariable logistic regression analysis was performed for risk factors. Subsequently, we also performed a multivariable logistic regression model, including the significant factors in the univariable models. All head and neck procedures (n = 19) were classified as non-adherent to SAP, primarily due to deviations in antibiotic choice and duration reflecting procedure-specific prophylaxis practices. Because SAP adherence showed no variability within this subgroup, inclusion of head and neck procedures in the multivariable model would have resulted in model instability and unreliable parameter estimates. Therefore, these procedures were excluded from the multivariable analysis. All statistical analyses were conducted using the R software for statistical computing version 4.5.1 (R Core Team, R Foundation for Statistical Computing, Vienna, Austria). Two-sided *P*-values ≤0.05 were considered statistically significant.

## Results

During the study period, 261 surgical procedures were identified and included in the analysis. Out of the total encounters, 79.7% were female, the mean age (SD) was 52.1 (±15.1), the mean weight was 71.44 (±16.08) kg. Breast procedures were half of the procedures done with 55.9%, followed by general surgery (21.5%). Table 1 summarizes the demographics and clinical characteristics of the surgical encounters stratified by overall adherence.

**Table 1:** Baseline demographics and clinical characteristics of patients.

Variables	No. (%)		
	All patients (N = 261)	Overall adherent (N = 172)	Non-adherent (N = 89)
**Age, mean (SD), years**	52.1 (15.1)	52.3 (14.1)	51.8 (17.0)
**Weight, mean (SD), kg**	71.4 (16.1)	70.0 (15.8)	74.3 (16.4)
**Gender**			
Female	208	145 (69.7%)	63 (30.3%)
Male	53	27 (50.9%)	26 (49.1%)
**Procedure type**			
Breast surgery	146	119 (81.5%)	27 (18.5%)
General surgery	56	34 (60.7%)	22 (39.3%)
Gyne-oncology	23	9 (39.1%)	14 (60.9%)
Urology	17	10 (58.8%)	7 (41.2%)
Head and neck procedures	19	0 (0%)	19(100%)
**ASA score**			
1	16	12 (75%)	4 (25%)
2	229	150 (65.5%)	79 (34.5%)
3	16	10 (62.5%)	6 (37.5%)
**Surgery period, mean (SD), minutes**	188.2 (132.8)	160.4 (128.4)	242.6 (125)
**Wound classification**			
Clean	173	127 (73.4%)	46 (26.6%)
Clean-contaminated	86	45 (52.3%)	41 (47.7%)
Contaminated	2	0 (0%)	2 (100%)
**Type of surgery**			
Elective	259	171 (66%)	88 (34%)
Urgent	2	1 (50%)	1 (50%)

Out of the 261 surgical encounters, 172 (65.9%) were adherent to the guidelines overall. Regarding the four individual metrics, the adherence was as follows: adherence to the antibiotic choice 216 (82.8%), adherence to weight-based dosing adjustment: 257 (99.6 %), adherence to re-dosing: 56 out of 70 (80%), and adherence to duration: 219 (84.6 %) (Table 2).

**Table 2:** Antibiotic guideline adherence for each individual metric.

Metrics	No. (%)		
	All patients, No.	Guideline adherent	Guideline nonadherent
Overall	261	172 (65.9%)	89 (34.1%)
Choice of antibiotic	261	216 (82.8 %)	45 (17.2 %)
Weight-based adjustment	258	257 (99.6 %)	1 (0.4 %)
Re-dosing^a^	70	56 (80 %)	14 (20%)
Duration (<24 hours)	259	219 (84.6 %)	40 (15.4 %)

a For re-dosing only surgeries who required re-dosing were considered for evaluation.

The univariable regression analyses identified several factors significantly associated with non-adherence to the SAP protocol, including gender, weight, procedure type, surgery time, and wound classification (Table 3). In the multivariable logistic regression analysis, procedure type and procedure duration remained significantly associated with the non-adherence. Specifically, the adjusted odds ratio (aOR) was 1.35 (95% confidence interval [CI], 1.16-1.58) for surgery period and 7.76 (95% CI, 2.13-30.86) for gyne-oncology procedures compared with general surgery procedures (Table 3). Notably, in the multivariable analysis, we elected to exclude head and neck procedures since all cases were non-adherent to the guidelines, and to merge contaminated with clean-contaminated surgeries given the very small number of contaminated cases (n = 2). Among the study population, the cumulative incidence of SSIs was 8.4%. Of those who developed an SSI, the majority were organ/space infections (52.0%), followed by deep incisional SSIs (33.3%) and superficial incisional SSIs (14.0%). Non-adherent cases were more likely to have SSI compared to adherent (15.7% vs 4.7%, *P* = 0.004). Among patients who developed SSI, non-adherence was most commonly observed in antimicrobial choice, with eight of 22 cases (36.4%), and in prolonged prophylaxis duration beyond 24 hours, occurring in eight of 21 cases (38.1%).

**Table 3:** Univariable and multivariable logistic regression with OR of non-adherence, including.

Variables	Univariable OR (95% CI)	*P*-value	Multivariable OR (95% CI)	*P*-value
Age (per year)	1.00 (0.98-1.01)	0.80	–	–
Gender				
Male	Ref		Ref	
Female	0.45 (0.24-0.84)	0.011	0.45 (0.16-1.20)	0.12
Weight (per kg)	1.02 (1.00-1.03)	0.041	1.01 (0.99-1.03)	0.33
Surgery period	1.32 (1.17-1.51)	<0.0001	1.35 (1.16-1.58)	**0.0001**
Procedure type				
General surgery	Ref		Ref	
Breast surgery	0.35 (0.18-0.69)	0.0025	3.91 (0.77-23.84)	0.12
Gyne-oncology	2.40 (0.90-6.69)	0.08	7.76 (2.13-30.86)	**0.002**
Urology	1.08 (0.35-3.25)	0.89	1.94 (0.54-7.05)	0.31
Wound classification				
Clean	Ref		Ref	
Clean-contaminated / contaminated	2.64 (1.55-4.53)	0.0004	3.39 (0.87-15.26)	0.09

CI, confidence interval; OR, odds ratios; Ref, reference.

Significant variables in the univariable analyses.

## Discussion

In our single-cohort analysis, we identified a suboptimal adherence rate of 65% to institutional SAP guidelines. While weight-based dosing was appropriately applied in most procedures, non-adherence was observed in other metrics, including antibiotic selection, timely intraoperative re-dosing, and discontinuation of prophylaxis within 24 hours.

The reported adherence rates to SAP guidelines in the literature vary considerably, ranging from 9% to as high as 70% [[4], [6], [7]]. Bardia *et al.* reported an overall adherence of 64.1% in a multicenter cohort analysis, with high compliance for timing of the first dose (99.4%) but lower adherence for intraoperative re-dosing (73.2%) [6]. Similarly, an Italian hospital demonstrated notable improvements following the implementation of stewardship interventions, with overall adherence increasing from 36.6% to 57.9% [8]. Significant improvements were documented across multiple domains, including indication, antibiotic selection, and appropriate duration of prophylaxis. In contrast, studies from low- and middle-income countries have consistently shown lower compliance rates, often below 50% and in some cases as low as 9% [[7], [9]]. Taken together, our findings place the adherence rate within the higher range of reported values, while still highlighting clear opportunities for targeted improvement in re-dosing practices, antibiotic choice, and prophylaxis duration.

Several factors have been identified as contributors to non-adherence, including the type of surgery, higher American Society of Anesthesiologists (ASA) score, urgency of the operation, prolonged operative duration, surgeon’s level of experience, procedures performed outside regular working hours, and the absence of clear institutional guidelines [[4], [10], [11]]. In our study, longer operative duration emerged as an independent risk factor for non-adherence, highlighting the need to prioritize ASP interventions for surgical procedures with prolonged duration.

Several surgical procedures were linked to pre-operative non-adherence, including gyne-oncology, orthopedic and neurosurgeries [[12], [13]]. In our cohort, gyne-oncological procedures were associated with higher odds to nonadherence compared to general oncologic procedures. Because all head and neck procedures demonstrated uniform non-adherence and were therefore excluded from the multivariable analysis, the adjusted findings primarily reflect non-head and neck oncological procedures, and caution is warranted when extrapolating these results to head and neck surgery.

Similar to prior studies, non-adherence to SAP was associated with higher rates of SSI. However, this finding should be interpreted with caution. Given the retrospective study design and the close inter-relationship between SAP non-adherence, procedure type, and operative duration, causal inference cannot be established. The observed association may therefore reflect underlying surgical complexity rather than the independent effect of SAP non-adherence alone. Multivariable analysis was not performed because of the limited sample size and insufficient statistical power.

Our study has several limitations. First, as a retrospective observational study, it is subject to inherent methodological constraints; however, a clear and standardized approach to data extraction was applied to minimize bias. Secondly, our sample size is relatively small, which might reduce the statistical power to identify certain predictors. Also, our study has limited generalizability to non-oncology settings. Finally, we did not evaluate specific human factors that may influence adherence, such as the surgeon’s years of experience, level of training, or whether the surgery was performed during or outside of regular working hours.

Despite these limitations, our study provides important insight into SAP non-adherence across a range of oncological surgical procedures. These findings highlight the need for strengthened stewardship strategies, with particular emphasis on optimizing intraoperative re-dosing, antibiotic selection, and prophylaxis duration, to improve compliance with guidelines.

## Declaration of competing interest

The authors have no competing interests to declare.

## References

[1] D’Ancona F.P., Claudia I. (2024). *Infections in Surgery: Prevention and Management*.

[2] Mahdi H., Gojayev A., Buechel M., Knight J., SanMarco J., Lockhart D. (2014). Surgical site infection in women undergoing surgery for gynecologic cancer. Int J Gynecol Cancer.

[3] Dhole S., Mahakalkar C., Kshirsagar S., Bhargava A. (2023). Antibiotic prophylaxis in surgery: current insights and future directions for surgical site infection prevention. Cureus.

[4] Berrondo C., Carone M., Katz C., Kenny A. (2022). Adherence to perioperative antibiotic prophylaxis recommendations and its impact on postoperative surgical site infections. Cureus.

[5] Bratzler D.W., Dellinger E.P., Olsen K.M., Perl T.M., Auwaerter P.G., Bolon M.K. (2013). Clinical practice guidelines for antimicrobial prophylaxis in surgery. Am J Health Syst Pharm.

[6] Bardia A., Treggiari M.M., Michel G., Dai F., Tickoo M., Wai M. (2021). Adherence to guidelines for the administration of intraoperative antibiotics in a nationwide US sample. JAMA Netw Open.

[7] Ali N., Hamouda R., Tarek R., Abdelhamid M., Lashin A., Hassan R. (2025). Non-adherence to surgical antibiotic prophylaxis guidelines: findings from a mixed-methods study in a developing country. Antimicrob Resist Infect Control.

[8] Segala F.V., Murri R., Taddei E., Giovannenze F., Del Vecchio P., Birocchi E. (2020). Antibiotic appropriateness and adherence to local guidelines in perioperative prophylaxis: results from an antimicrobial stewardship intervention. Antimicrob Resist Infect Control.

[9] Kıymaz YÇ, Karakök T., Büyükkörük M., Manavlı B., Baysal C., Karaşın M.F. (2025). Evaluation of surgical antimicrobial prophylaxis compliance: a multicenter point prevalence study. Am J Infect Control.

[10] Hassan S., Chan V., Stevens J., Stupans I. (2021). Factors that influence adherence to surgical antimicrobial prophylaxis (SAP) guidelines: a systematic review. Syst Rev.

[11] Muller A., Leroy J., Hénon T., Patry I., Samain E., Chirouze C., Bertrand X. (2015). Surgical antibiotic prophylaxis compliance in a university hospital. Anaesth Crit Care Pain Med.

[12] Kosić M., Miljković B., Barišić V., Vezmar-Kovačević S., Jovanović M., Vučićević K. (2025). Antimicrobial prophylaxis in elective orthopaedic surgical procedures at the tertiary care hospital: a prospective observational study. Antibiotics (Basel).

[13] Pereira L.B., Feliciano C.S., Bellissimo-Rodrigues F., Pereira LRL. (2024). Evaluation of the adherence to surgical antibiotic prophylaxis recommendations and associated factors in a University Hospital: a cross-sectional study. Am J Infect Control.

